# Academic Achievement in Language and Mathematics: The Role of Cognitive Abilities and Academic Self-Concept Across the Third Cycle and Secondary Education

**DOI:** 10.3390/jintelligence14040057

**Published:** 2026-04-01

**Authors:** Leandro S. Almeida, Gina C. Lemos, Ana Cristina Silva, Francisco Peixoto

**Affiliations:** 1 CIPsi—Psychology Research Center, School of Psychology, University of Minho, 4710-057 Braga, Portugal; leandro@psi.uminho.pt; 2 Instituto Politécnico de Setúbal, Escola Superior de Educação, 2910-761 Setúbal, Portugal; 3 CIEd—Research Centre on Education, Institute of Education, University of Minho, 4710-057 Braga, Portugal; 4 Interdisciplinary Research Centre in Education (EDUNOVA.ISPA), ISPA—Instituto Universitário, 1149-041 Lisbon, Portugal

**Keywords:** intelligence, cognitive ability, self-concept, academic achievement, mathematics achievement, language achievement

## Abstract

Research on academic achievement highlights the combined role of cognitive abilities and motivational beliefs. Grounded in the CHC framework, this study examined how three broad cognitive abilities—verbal, numeric, and spatial—and academic self-concept jointly predict achievement in Portuguese and mathematics. A sample of 3034 students from the third cycle (grades 7–9) and secondary education (grades 10–12) completed the BAC-AB cognitive battery and a validated academic self-concept scale. Using multigroup structural equation modelling, we tested whether the predictive patterns differed across educational stages. Academic self-concept emerged as the most consistent predictor across subjects and levels. Cognitive contributions displayed clear developmental differentiation: verbal ability was more strongly associated with Portuguese (and increasingly with Mathematics) in secondary education, whereas numeric and spatial abilities were comparatively more relevant for Mathematics in the third cycle. These patterns support the view that linguistic, quantitative, and visuospatial processes contribute to achievement in distinct and developmentally sensitive ways. Overall, the findings underscore the importance of instructional approaches that build on quantitative and spatial strengths in earlier grades while progressively supporting advanced verbal comprehension and reasoning in later schooling.

## 1. Introduction

In recent decades, academic success has increasingly been conceptualised as a dynamic process rather than merely an outcome. Beyond positive results, a comprehensive view includes learning skills, student satisfaction, and well-being, as well as skill transferability to future educational and professional contexts (Anghel, 2023; Gottfried & Plasman, 2018). Within compulsory education, two curricular domains are foundational: the language of instruction and mathematics. Competence in the former (morphological, semantic, syntactic) and the latter (calculation, geometry, problem solving) underpins academic, civic, and professional learning, directly influencing achievement levels.

Academic achievement can be framed within Bronfenbrenner’s bioecological PPCT model as the product of proximal processes; that is, recurring and increasingly complex interactions through which students engage with caregivers, teachers, peers, and learning materials (Bronfenbrenner, 2005; Bronfenbrenner & Morris, 2006). The impact of these everyday learning processes depends on person characteristics such as motivational dispositions, cognitive resources, and self-beliefs; the contexts in which they occur, including in the family, classroom, school, and broader sociocultural situations; and time, namely, their frequency and duration, developmental timing, and the historical period. From this perspective, achievement reflects not only individual and contextual inputs but also how these inputs shape the quality, stability, and continuity of students’ sustained engagement with learning opportunities. In this study, we focus on person-level factors (cognitive abilities and academic self-concept) while controlling key sociodemographic characteristics, thus isolating their unique associations with achievement within relevant ecological contexts.

Accordingly, focusing on adolescents in the final years of basic education (third cycle; grades 7–9) and in secondary education (grades 10–12), we examine the extent to which broad cognitive abilities and academic self-concept jointly predict grades in Portuguese and mathematics among Portuguese students. In the present study, we focus on academic achievement, operationalised through students’ grades, as a core dimension of performance in these foundational domains.

### 1.1. Sociodemographic Factors and Academic Achievement

Academic (un)success is widely recognised as a complex and multidetermined phenomenon shaped by the interaction of numerous personal, familial, school, and broader contextual influences. Recent systematic reviews describe academic achievement as emerging from a constellation of interrelated variables spanning cognitive, motivational, psychological, socioeconomic, sociocultural, and institutional domains, underscoring its inherent complexity (A. Costa et al., 2024; Yigiter, 2025). Within this multifaceted landscape, the literature consistently highlights the role of sociodemographic factors as among the most influential predictors of students’ educational trajectories. These include parental educational level, socioeconomic status, gender, and other markers of social position, which shape the conditions under which children and adolescents learn and develop (A. Costa et al., 2024; Yigiter, 2025).

Among these, students’ sociocultural and family backgrounds play a particularly key role throughout education, as they shape the conditions under which children and adolescents learn and develop. Research consistently indicates that parents’ educational level, especially that of mothers, is one of the most significant variables explaining differences in students’ learning outcomes (Alves et al., 2016; Harding, 2015; Magnuson, 2007). Highly educated parents are more involved and supportive in school matters; for example, they help children with school tasks and are more engaged in their children’s academic life (Tan et al., 2019). Student gender is also frequently examined in relation to academic performance, with female students generally demonstrating higher levels of classroom engagement and participation, as well as greater motivation to complete academic tasks and learn (M. Carroll, 2023; Voyer & Voyer, 2014).

Using two large-scale samples of ninth-grade students, Johannsen et al. (2024) examined how gender, parental socioeconomic status (SES), and immigrant background relate to academic achievement in language and mathematics, after accounting for psychological predictors. Their findings showed consistent domain-specific patterns: girls tended to achieve higher grades and test scores in German, whereas boys outperformed girls on mathematics test scores, despite girls reporting higher mathematics grades. Parental SES emerged as a particularly robust predictor, with higher SES consistently associated with better academic outcomes, especially in language achievement across both grades and standardised test scores.

### 1.2. Cognitive Abilities and Academic Achievement

In what concerns psychological variables, a substantial body of research underscores the relevance of cognitive and motivational factors in explaining academic success (Deary et al., 2007; Kaya et al., 2015; Lemos et al., 2025; Valentini & Laros, 2014). From a cognitive perspective, in addition to basic cognitive functions such as attention and memory, research increasingly emphasises the role of executive and metacognitive functions (Coutinho et al., 2005; Santoso et al., 2019). These functions are closely related to learning and metacognitive strategies, which are characteristic of more active, deep, and self-regulated forms of learning. In other words, learning involves the mobilisation of multiple cognitive functions and abilities, which are integrated within the broad concept of intelligence (Colom & Flores-Mendoza, 2007; Primi et al., 2010; Valentini & Laros, 2014).

When examining the cognitive dimension, particularly the concept of intelligence, it is essential to recognise the ongoing debate regarding how it should be defined. The idea that diverse cognitive abilities converge into a single general intelligence factor has a long-standing tradition in psychology (Lemos et al., 2020; Lubinski, 2004). Classical intelligence quotient (IQ) tests were built upon this assumption, positing that a variety of abilities arising from different cognitive processes and task domains, such as verbal, numerical, and spatial abilities, could be meaningfully combined into an overall score representing general intellectual ability. This perspective tended to downplay the specificity and relative independence of distinct cognitive abilities, an issue that becomes particularly evident in adulthood, where intellectual profiles diverge as a result of individuals’ academic and professional experiences as well as their personal interests and investment patterns (Ackerman, 1996; Anastasi, 1983; Cattell, 1987). A middle-ground position emerged toward the end of the twentieth century with the development of the CHC (Cattell–Horn–Carroll) hierarchical model. This framework proposes that intercorrelations among nearly one hundred primary cognitive abilities can be organised into roughly a dozen broad second-order factors, which themselves intercorrelate and converge into a higher-order general factor (J. B. Carroll, 1993, 1997). The CHC model has gained substantial support in the literature and is widely used both for evaluating existing test batteries and as a theoretical foundation for developing new assessment instruments (Lemos et al., 2020; McGrew & Flanagan, 1997; Schneider & McGrew, 2018). However, several scholars have argued that the model primarily serves to classify intelligence tests rather than define intelligence in terms of underlying cognitive or neurological processes (Canivez & Youngstrom, 2019; Kovacs & Conway, 2016).

Research on academic achievement within the CHC framework shows that general intelligence (g) tends to exhibit the strongest association with academic achievement, whereas associations with specific cognitive abilities are more variable in strength depending on the particular school subject and the specific ability assessed (Benson et al., 2016; Caemmerer et al., 2018; Hajovsky et al., 2025; Lemos et al., 2025; McGrew & Wendling, 2010; Roth et al., 2015; Zaboski et al., 2018). Across this literature, CHC broad abilities show smaller and more differentiated relations once the influence of g is properly modelled (e.g., using higher-order vs. bifactor approaches) (Benson et al., 2016; Zaboski et al., 2018). In turn, verbal ability, typically operationalised as comprehension knowledge (Gc), emerges as the most robust and consistent specific correlate of achievement overall, with particularly clear links to literacy outcomes (e.g., basic reading skills, reading fluency, reading comprehension, spelling, and writing performance) and more modest but meaningful links to mathematics when tasks carry substantial language demands (Benson et al., 2016; Caemmerer et al., 2018; Hajovsky et al., 2025; Zaboski et al., 2018).

A comprehensive understanding of the relationship between cognitive abilities and academic achievement benefits from examining multiple intelligence and achievement measures simultaneously, allowing the assessment of how different cognitive processes contribute to reading across proficiency levels. Meta-analytic evidence shows that broad cognitive abilities, particularly comprehension knowledge and auditory processing, reliably predict foundational and advanced reading skills, with basic reading serving as an important precursor to higher-level comprehension (Hajovsky et al., 2025). Reading comprehension can be conceptualised as the gradual construction of a mental representation of the text, emerging from the continuous integration of new textual information with relevant prior knowledge, supporting both local and global coherence (van den Broek & Kendeou, 2022). Converging results from Hajovsky et al. (2014, 2025) further show that broad cognitive capacities, including comprehension knowledge and working memory, contribute to reading comprehension through their role in supporting basic decoding and linguistic processes.

In mathematics, the most prominent CHC correlate is typically numerical/quantitative reasoning, frequently captured by fluid reasoning (Gf) and, when assessed, quantitative knowledge components; these abilities show consistent relations with both calculation and, particularly, mathematical problem solving (Caemmerer et al., 2018; McGrew & Wendling, 2010). Spatial ability (Gv) tends to show limited and inconsistent relations with language outcomes, but it is more clearly implicated in mathematics—particularly in tasks involving reasoning, problem solving, and spatially loaded content—although its effects are generally smaller than those of g and can be sensitive to whether g is adequately controlled (Benson et al., 2016; Caemmerer et al., 2018).

Developmental evidence indicates that these relations are not static across age and schooling. Meta-analytic and synthetic reviews suggest that the relative importance of *g* and specific CHC abilities changes with increasing educational demands: in reading, the contribution of *g* and particularly Gc tends to increase with age, consistent with the growing role of vocabulary, background knowledge, and language-based comprehension processes in later schooling (Hajovsky et al., 2025; McGrew & Wendling, 2010; Zaboski et al., 2018). In mathematics, numerical/quantitative reasoning shows comparatively stable relations across development, remaining relevant from early schooling through adolescence, particularly for higher-order mathematical reasoning, while the role of *g* may be relatively stronger for more basic math outcomes at earlier ages and less dominant later on (McGrew & Wendling, 2010; Zaboski et al., 2018). Evidence also suggests that the contribution of Gc to mathematics may become more visible with age as mathematical work increasingly involves linguistic comprehension (e.g., verbal problems and instructionally embedded tasks) (Caemmerer et al., 2018; McGrew & Wendling, 2010). Across domains, a recurrent methodological conclusion is that estimates of “specific ability” effects can be inflated when *g* is not explicitly accounted for; therefore, clarifying the unique roles of verbal (Gc), numerical/quantitative (Gf/Gq), and spatial (Gv) abilities—especially their developmental shifts—depends strongly on using models that separate general and specific variance (Benson et al., 2016; Zaboski et al., 2018).

Although the CHC framework provides a comprehensive and empirically well-supported structure for organising cognitive abilities, it remains primarily a psychometric model. It reflects patterns of covariation among test scores rather than offering a process-based or developmental explanation of how cognition functions or changes over time (J. B. Carroll, 1993; Schneider & McGrew, 2018). Consequently, the framework does not clarify how specific abilities contribute to learning, nor does it account for the ways in which instructional experiences may influence cognitive development. Moreover, several studies indicate that the apparent contribution of broad abilities can vary substantially depending on how the model is specified. For example, different analytic approaches such as higher-order versus bifactor models often produce shifts in the relative importance of domain-specific abilities, with many of these effects diminishing once variance attributed to general intelligence is properly controlled (Benson et al., 2016; Kan et al., 2024; Zaboski et al., 2018). Taken together, these considerations highlight the need for caution when interpreting CHC-based findings in educational contexts, particularly when drawing domain-specific implications for teaching and learning.

### 1.3. Academic Self-Concept and Academic Achievement

Alongside cognitive abilities, motivational variables gained increasing prominence in the second half of the twentieth century due to their substantial contribution to learning quality, academic performance, and overall academic success (Steinmayr et al., 2018). Beyond the traditional distinction between intrinsic and extrinsic motivation, more operational and empirically grounded conceptualisations of motivation—particularly academic motivation—have emerged. These include constructs such as perceptions of competence and self-efficacy, self-concept, and self-esteem, as well as learning goals and achievement orientations.

Within this framework, self-concept—and especially academic self-concept—has received considerable attention because of its influence on performance in specific domains of knowledge, such as mathematics and verbal abilities. Often conceptualised as a mediating variable, self-concept plays a crucial role across multiple contexts, including academic, athletic, and professional domains (Fabriz et al., 2021; Gasparotto et al., 2018; Marsh, 2023; Marsh et al., 2015; Pipa & Peixoto, 2014). Defined as individuals’ perceptions of themselves, self-concept has been extensively studied with respect to its structural properties and, within educational psychology, its associations with academic achievement and related outcomes, such as vocational aspirations, academic adjustment, and academic engagement (Jin et al., 2025; Sinclair et al., 2019; van der Aar et al., 2019; Wallace & Walker, 1990). In general, students tend to achieve better outcomes when they perceive themselves as competent, feel confident in their abilities, and maintain a positive academic self-concept. Empirical evidence further suggests that students with a high self-concept are more likely to align their behaviour and performance with their self-perceptions (Perinelli et al., 2022; Sherman et al., 2013) and engage more strongly in self-regulatory processes and persistent learning behaviours (Marsh & Martin, 2011).

A substantial body of longitudinal research has demonstrated that prior academic self-concept positively predicts subsequent academic performance, both school grades and standardised test scores, beyond what can be explained by prior achievement alone (Marsh & O’Mara, 2008; Sticca et al., 2023; Valentine et al., 2004). In a review of 56 longitudinal studies, (Valentine et al., 2004) showed that self-related beliefs, including self-concept and self-efficacy, retained a significant effect on academic outcomes even after controlling for previous performance. These effects are particularly pronounced when domain-specific dimensions of self-concept, such as verbal and mathematical self-concept, are examined in relation to corresponding academic domains (Huang, 2011; Möller et al., 2009, 2020). Moreover, the association between academic self-concept and performance has consistently been characterised as reciprocal, with self-concept influencing achievement and achievement, in turn, shaping self-concept over time (Huang, 2011; Möller et al., 2011; Niepel et al., 2014; Wu et al., 2021). At the same time, evidence suggests that the magnitude of the self-concept–achievement link depends on the type of performance indicator used, with studies relying on standardised test scores generally reporting weaker associations than those using teacher-assigned grades, likely reflecting differences in evaluative criteria and contextualised feedback processes (Marsh et al., 2016; Wu et al., 2021).

In addition, research has documented systematic developmental changes in academic self-concept across the school years. Several studies report a general decline in academic self-concept during adolescence, particularly during the third cycle of basic education (i.e., lower secondary education or junior high school) (Green et al., 2012; Preckel et al., 2013). Skaalvik and Valås (1999) proposed that the relationship between self-concept and academic performance is age-dependent, being more clearly supported in primary school and becoming less consistent in early adolescence. Other authors have suggested that academic self-concept is increasingly shaped by cumulative experiences of success and failure, as well as by social comparison processes and external standards of achievement (Helmke & van Aken, 1995; Marsh & Craven, 2006). With increasing age, students’ academic self-concepts tend to become more differentiated, realistic, and closely aligned with objective indicators of performance. Supporting this view, Marsh et al. (1998) showed that the reliability, stability, and factorial validity of academic self-concept measures improve across development, indicating greater differentiation and temporal stability over time.

These issues are especially salient when considering the relative contributions of academic self-concept and intelligence to achievement in the language of instruction and mathematics. In particular, examining domain-specific cognitive abilities (e.g., verbal and numerical) alongside corresponding academic self-concepts can provide a more nuanced account of academic performance, given that cognitive and motivational factors contribute both uniquely and interactively to achievement outcomes. In a large sample of Flemish seventh-grade students, Lavrijsen et al. (2022) found that intelligence was the strongest predictor of academic achievement. Nevertheless, motivational variables explained a substantial proportion of variance even after controlling for intelligence and personality, with academic self-concept emerging as the most relevant motivational predictor. Taken together, these findings underscore the importance of examining the interplay between cognitive abilities and academic self-concept across different stages of schooling, particularly when considering domain-specific achievement in language and mathematics.

### 1.4. Current Study

Anchored in Bronfenbrenner’s bioecological PPCT framework (Bronfenbrenner, 2005; Bronfenbrenner & Morris, 2006), we conceptualise academic achievement as the outcome of proximal processes, that is, recurring and increasingly complex interactions that students sustain with teachers, peers, and curricular materials, the effectiveness of which depends on person characteristics (e.g., cognitive abilities and academic self-concept), context (family, school, and wider sociocultural conditions), and time (frequency, duration, and developmental timing). In the present study, we focus on person-level factors (broad cognitive abilities (verbal, numeric, spatial) and academic self-concept) and test their joint associations with grades in Portuguese and mathematics, while controlling sociodemographic constraints (gender and mother’s education/SES) that shape opportunity structures and learning routines.

Converging evidence from CHC-based syntheses and cross-battery analyses indicates that cognition–achievement relations exhibit developmental differentiation across schooling, with broad abilities relating to achievement in domain-specific and age-sensitive ways (for example, shifts in the relative weights of verbal/Gc, numeric/Gf, and spatial/Gv from early to later grades; McGrew & Wendling, 2010; Zaboski et al., 2018). In particular, studies show that numeric and spatial abilities tend to display stronger links to mathematics when curricular content is more procedural or representational, whereas verbal or comprehension knowledge (Gc) becomes more salient in later stages, with robust effects on language-loaded outcomes and increasing contribution when mathematical tasks are text- and discourse-heavy (Caemmerer et al., 2018). Considering Portuguese upper-secondary curriculum guidance, which emphasises integrated competencies and more complex textual processing in both Portuguese and mathematics (Direção-Geral da Educação, 2018a, 2018b; Eurydice, 2026), we expect differences in the effects of specific cognitive abilities on academic achievement across subjects, as well as differences between younger (third cycle) and older (secondary education) students (McGrew & Wendling, 2010; Caemmerer et al., 2018; Zaboski et al., 2018).

Beyond cognitive abilities, we examine the extent to which academic self-concept predicts achievement in both subjects across school levels. Consistent with prior research, we expect academic self-concept to explain additional variance in achievement for Portuguese and mathematics beyond cognitive abilities (Lavrijsen et al., 2022). This hypothesis is reinforced by meta-analytic evidence showing robust links between academic self-concept and achievement with age-related moderation effects (Möller et al., 2020; Wu et al., 2021).

Finally, given consistent evidence that sociodemographic factors are associated with both cognitive development and school outcomes, we include student gender and mother’s educational level as covariates and examine their effects on achievement, cognitive abilities, and academic self-concept within each school cycle. This allows us not only to adjust the focal cognitive–motivational relations for background influences but also to provide a more comprehensive account of how structural inequalities and gender-linked patterns co-occur with cognitive performance, self-beliefs, and achievement across adolescence.

## 2. Materials and Methods

This study adopted a cross-sectional, observational design with two educational groups corresponding to adjacent levels of schooling (3rd cycle, grades 7–9; secondary education, grades 10–12).

### 2.1. Participants

Participants were 3034 Portuguese students from the 3rd cycle of basic schooling (7th to 9th grade, *N* = 1358) and secondary education (10th to 12th grade, *N* = 1676) attending public schools. Of the total sample, 51.6% were female, and ages ranged from 12 to 19 years (*M* = 14.91, *SD* = 1.79). Regarding mothers’ educational level, 5.7% of students had mothers with primary education, 24.5% with middle school education, 29.2% with secondary education, and 40.7% with higher education.

School selection followed a stratified procedure. Public schools were first grouped by NUT II region (North, Centre, Lisbon and Tagus Valley, Alentejo, Algarve, Madeira, and the Azores), ensuring geographical coverage. Within each region, schools were then stratified using the Ministry of Education’s official socio-educational categorisation by urban–rural location. Schools classified as Priority Intervention Educational Territories (TEIP) were not included due to their highly specific sociodemographic profile and targeted compensatory measures. Participant inclusion required the availability of complete cognitive, academic self-concept, and administrative achievement data for the year of assessment. Students with missing data on any of these components were excluded.

In the Portuguese education system, basic schooling comprises grades 1–9 and is subdivided into three cycles. The present study focuses specifically on the 3rd cycle (grades 7–9). Secondary education comprises grades 10–12. Occasional references to “primary,” “middle,” and “secondary” schooling follow international conventions that map onto the 1st cycle (primary), the 2nd and 3rd cycles (middle/lower secondary), and upper secondary education, respectively.

### 2.2. Measures

#### 2.2.1. Cognitive Abilities

Cognitive abilities were evaluated using “Bateria de Aptidões Cognitivas” (BAC-AB; Lemos & Almeida, 2015), a standardised instrument designed to measure three core domains of intellectual functioning—verbal, spatial and numerical—across three progressively complex cognitive processes: comprehension, reasoning, and problem solving.

The BAC-AB comprises nine timed subtests, each targeting specific cognitive abilities within these domains. Verbal ability is assessed through the synonyms, analogies, and expressions subtests; spatial ability through the figure rotation, cubes sequences, and movements and shapes subtests; and numerical ability through the calculus, numeric sequences, and problems subtests. Administration times vary by subtest, ranging from four to fifteen minutes.

**Figure 1:**
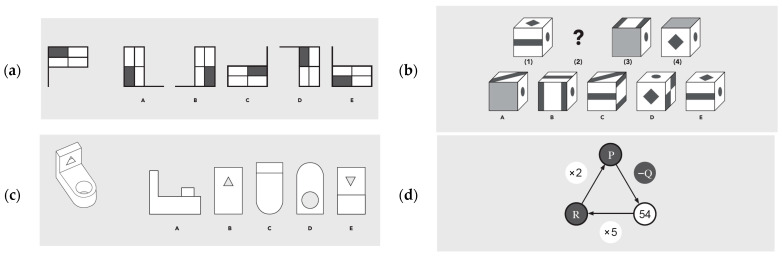
Example items from the administered intelligence battery. Figure rotation example item (**a**). Cubes sequences example item (**b**). Movements and shapes example item (**c**). (Which option (A, B, C, D, E) corresponds to a view of the figure shown ?) Calculus example item (**d**).

Verbal ability is assessed through three subtests: the synonyms subtest includes twenty-four items and is administered in four minutes; it assesses lexical knowledge and semantic relationships (e.g., The group scout. A. Lookout B. Analyst C. Coordinator D. Broker E. Director). The analogies subtest consists of twenty-four verbal analogy items, also with a four-minute time limit, and measures verbal reasoning (e.g., Foot is to ___________ as shoe is to glove. A. Finger B. Ring C. Arm D. Sock E. Hand). The expressions subtest includes twelve items involving idiomatic and figurative language, with six minutes for completion (e.g., Rita gets up with the chickens. A. Rita sleeps near a chicken coop. B. Rita hears the cackling of chickens. C. Rita is an early riser. D. Rita likes to sing when she wakes up. E. Rita usually wakes up early.). Spatial ability is assessed through three subtests: figures rotation, which comprises twenty mental rotation tasks and is administered in seven minutes (e.g., Figure 1a); cubes sequences, with twenty items involving spatial orientation and cube rotation, with ten minutes of administration time (e.g., Figure 1b); and movements and shapes, which includes twenty spatial and mechanical problem-solving items, with twelve minutes for completion (e.g., Figure 1c). Numerical ability is measured through three subtests: calculus, consisting of eight calculation items and administered in ten minutes (e.g., Figure 1d); numeric sequences, which includes fifteen items assessing numerical reasoning, with a ten-minute time limit (e.g., 2 1 3 1 P 1 5 Q 6); and problems, comprising twelve applied numerical problem-solving situations, completed in fifteen minutes (e.g., There are 57 ladies and 39 men on a dance floor. Each of the men made a pair with a lady. The other ladies formed pairs with one another. (a) How many pairs of people were on the dance floor? (b) How many pairs were formed only by ladies?).

Students enrolled in the 3rd cycle of basic education completed the version intended for seventh to ninth grades (BAC-A), whereas those attending secondary education completed the version designed for tenth to twelfth grades (BAC-B). The BAC-AB incorporates anchor items to ensure comparability between versions; specifically, approximately half of the items are shared across both forms. To adjust difficulty levels, the simplest items from BAC-A were excluded from BAC-B, while the most challenging items from BAC-B were not included in BAC-A.

Previous research has provided robust evidence supporting the reliability and validity of the BAC-AB subtests across both versions of the battery. Internal consistency coefficients for BAC-A ranged between 0.70 for analogies and 0.88 for calculus, while for BAC-B, they varied from 0.82 for expressions to 0.93 for calculus. Criterion validity was examined through Pearson correlations between cognitive performance and academic achievement in different school subjects. The findings indicated (1) a significant positive relationship between scores on the cognitive subtests and school achievement in all grades considered and (2) that these associations were stronger and more consistent in the earlier grades, gradually decreasing in magnitude and becoming more variable from the ninth grade onward (Lemos & Almeida, 2019).

#### 2.2.2. Academic Self Concept

Academic self-concept was assessed using the School Competence subscale from the Self-Esteem and Self-Concept Scale developed by Peixoto and Almeida (1999, 2011). This subscale includes five items that assess students’ perceptions of their overall academic competence (e.g., “Some students understand everything their teachers explain in class”). Responses were given on a 4-point scale ranging from “Completely different from me” to “Exactly like me.”

#### 2.2.3. Academic Achievement

Academic achievement was assessed based on students’ grades in Portuguese language and mathematics. Within the Portuguese school system, during the 3rd cycle of basic education (corresponding to the seventh, eighth, and ninth grades), teachers evaluate students using a grading scale ranging from 1 to 5 points in each subject (1 and 2 = fail; 3 to 5 = pass). At the secondary education level (corresponding to the tenth, eleventh, and twelfth grades), teachers use a grading scale ranging from 1 to 20 points in each subject (1 to 9 = fail; 10 to 20 = pass). These data were collected from the schools’ administrative offices.

### 2.3. Procedure

All data collection procedures were conducted in compliance with ethical and legal standards. Prior authorisation was obtained from the National Data Protection Commission, the Directorate-General for Education of the Ministry of Education, and the Ethics Committee of the higher education institution responsible for this study. Before participation, students were informed about the objectives of the research, the voluntary nature of their involvement, and the confidentiality of their responses. Informed assent was provided by students following receipt of parental consent through formal letters.

The assessment protocol for this study included two main measures: academic self-concept and cognitive abilities. The self-concept measure was administered on the first day of data collection, prior to the cognitive subtests.

Administration of BAC-AB was carried out by psychologists specifically trained for this purpose. Standardised instructions were rigorously followed, including illustrative examples for each subtest. Data collection took place during regular class hours, with a maximum of twenty-eight students per session and with prior consent from teachers. To minimise fatigue, the nine BAC-AB subtests were distributed across two sessions of approximately 90 min each, scheduled within the same week or in consecutive weeks. The order of administration was fixed and structured to prevent consecutive subtests assessing the same domain (verbal, numerical, or spatial). On average, students completed the BAC-AB in about eighty minutes.

Official school records provided students’ grades in the relevant curricular subjects, ensuring accuracy and consistency in academic achievement data.

### 2.4. Data Analyses

For the BAC subtests, item scores were summed to obtain a total score for each subtest. For academic achievement, z-scores were computed separately for each educational level (3rd cycle vs. secondary), given that the grading scales differ (1–5 for 3rd cycle students and 1–20 for secondary education students).

Data were analysed using Structural Equation Modelling (SEM) with the Maximum Likelihood Robust (MLR) estimator implemented in Mplus 8.14. The MLR estimator provides parameter estimates and test statistics that are robust to violations of multivariate normality (L. K. Muthén & Muthén, 1998–2023). Because students are nested in classes, analyses were carried out under TYPE = COMPLEX, treating *Class* as the clustering unit to obtain cluster-robust standard errors and fit statistics.

Model fit was evaluated using the Comparative Fit Index (CFI), the Tucker–Lewis Index (TLI), the Root Mean Square Error of Approximation (RMSEA), and the Standardised Root Mean Square Residual (SRMR). Following commonly used guidelines, values of CFI and TLI ≥ 0.90 and RMSEA and SRMR ≤ 0.08 were considered indicative of acceptable model fit (Hu & Bentler, 1999; Schumacker & Lomax, 2016).

Because the goal was to compare models across educational levels, we tested measurement invariance between 3rd cycle and secondary education students. We evaluated configural invariance (same number and pattern of factors), metric invariance (factor loadings constrained equal), and scalar invariance (both factor loadings and intercepts constrained equal). Given the sensitivity of the chi-square test to sample size, decisions regarding invariance were based on changes in fit indices, with ΔCFI < 0.010 and ΔRMSEA < 0.015 indicating invariance (Chen, 2007).

The structural model included four latent factors—verbal, numeric, spatial, and academic self-concept—each specified using their respective observed indicators. Mother’s educational level and student gender were included as covariates to control for their effects. The model was first estimated in the total sample to verify overall model fit and parameter stability. Because our aim was to examine whether the predictive paths differed across educational levels, we subsequently conducted multi-group structural equation modelling (MG-SEM) using cycle (3rd cycle vs. secondary education) as the grouping variable (Kline, 2023; Byrne, 2012). To assess whether specific regression coefficients differed significantly between groups, we used two complementary approaches:

1. Labelled parameters in group-specific models, allowing all parameters to vary freely across groups,
2. Wald tests of parameter constraints via the MODEL TEST command, specifying constraints of the form

0 = *b*_C3_ − *b*_Sec_, (1)

which tested whether regression effects differed significantly between the 3rd cycle and secondary education groups (B. Muthén & Asparouhov, 2015). Additionally, *MODEL CONSTRAINT* was used to compute raw group differences, their standard errors, and corresponding z-statistics, providing interpretable effect-size comparisons across groups. These procedures are mathematically equivalent to comparing freely estimated versus constrained models but offer direct estimates and significance tests for each cross-group difference (L. K. Muthén & Muthén, 1998–2023).

## 3. Results

### 3.1. Descriptive Statistics, Reliabilities, and Correlations

Table 1 presents the reliabilities, descriptive statistics, and intercorrelations among all study variables for the two educational groups (third cycle and secondary education students). Reliability was acceptable for the cognitive measures based on the three subtest scores per cognitive factor (α = 0.73–0.77). Reliability for the academic self-concept scale was α = 0.78. As expected, secondary education students showed higher mean scores on the cognitive tasks and slight differences in academic achievement compared with third cycle students. At both educational levels, achievement in mathematics was lower than in Portuguese and displayed higher standard deviations, indicating greater variability in mathematics performance. In secondary education, the lower variability observed in cognitive scores may be related to selection processes, whereby students with lower cognitive abilities are more likely to enrol in vocational pathways. Across both school groups, numeric ability exhibited the highest standard deviation values, indicating greater dispersion of scores than verbal and spatial abilities. Concerning academic self-concept, means and standard deviations were largely similar across the two educational groups.

Correlation coefficients were largely consistent across groups and aligned with theoretical expectations. Achievement in Portuguese and mathematics showed moderate positive associations with all cognitive abilities. The strongest correlations were observed between mathematics achievement and numeric ability in the third cycle and between Portuguese achievement and verbal ability at both educational levels. Spatial ability showed weaker associations with achievement, particularly with Portuguese achievement in both cycles. As expected, numeric ability was more strongly related to mathematics achievement, especially among third cycle students. Academic self-concept was positively associated with both achievement outcomes and cognitive abilities in both groups, although these associations with cognitive factors were weaker among secondary school students.

Mother’s educational level was positively related to achievement and cognitive abilities. Regarding gender, girls showed higher Portuguese achievement at both educational levels, whereas boys scored higher on spatial and numeric abilities in both cycles. Overall, these correlational patterns support the distinct yet related nature of the constructs and justify their inclusion in subsequent multigroup structural equation models.

### 3.2. Measurement Model and Invariance Test

We evaluated the psychometric structure of the BAC subtests with two theoretically driven alternatives: a unidimensional model (general intelligence; g) and a three-factor CHC model with verbal, numeric, and spatial abilities, each specified by their three intended indicators. In both models, we retained a theoretically justified residual covariance, reflecting content overlap between the expressions and synonyms indicators in the verbal domain. In the full sample, the CHC model demonstrated superior global fit (χ^2^(56) = 333.1, *p* < .001; CFI = 0.958; TLI = 0.946; RMSEA = 0.057, 95% CI [0.051, 0.063]; SRMR = 0.054; AIC = 141,926.3; BIC = 142,239.2) relative to the unidimensional alternative (χ^2^(66) = 602.5, *p* < .001; CFI = 0.919; TLI = 0.912; RMSEA = 0.073, 95% CI [0.068, 0.079]; SRMR = 0.079; AIC = 142,376.8; BIC = 142,629.5), supporting the use of CHC as the primary measurement model in subsequent multi-group analyses.

The academic self-concept measure was specified as a single latent factor defined by its five items. The confirmatory factor analysis showed very good fit (χ^2^(5) = 36.8, *p* < .001; CFI = 0.984; TLI = 0.968; RMSEA = 0.053, 95% CI [0.038, 0.070]; SRMR = 0.020). This well-fitting measurement model was carried forward to the subsequent analyses.

We tested configural, metric, and scalar invariance for the CHC model (verbal/numeric/spatial) and the unidimensional g model. For both model families, changes from configural to metric met the defined criteria of ΔCFI < 0.010 and ΔRMSEA < 0.015 (Table 2). From metric to scalar, the deterioration was smaller for CHC and larger for g (ΔCFI exceeding 0.010 for g), reinforcing the selection of CHC as the preferred representation of cognitive abilities in this dataset. Accordingly, we carried the CHC measurement model forward to examine cross-group differences in structural relations to Portuguese and mathematics achievement. The academic self-concept factor showed minimal changes from configural to metric and from metric to scalar (Table 2), supporting scalar invariance across the third cycle and secondary education.

Conclusions regarding CHC vs. g remained unchanged when we perturbed the residual covariances (e.g., removing one residual covariance at a time), indicating that CHC’s superiority is not an artefact of the residual specification.

### 3.3. Structural Model

Following the analytic plan, we first estimated the overall structural model in the full sample to evaluate the proposed relations among the latent variables. The model showed an adequate fit to the data: χ^2^(109) 736.7, *p* < .001; CFI = 0.952; TLI = 0.933; RMSEA = 0.044 (90% CI [0.041, 0.047]); SRMR = 0.033. We subsequently estimated the multigroup structural model based on the educational cycle. This model exhibited a deterioration in fit (χ^2^(238) = 1089.9, *p* < .001; CFI = 0.935; TLI = 0.917; RMSEA = 0.049 (95% CI [0.046, 0.052]); SRMR = 0.045), suggesting potential differences in structural relations between third cycle and secondary education students. Even so, the multigroup model still met commonly accepted thresholds for acceptable fit (Hu & Bentler, 1999; Schumacker & Lomax, 2016).

**Figure 2:**
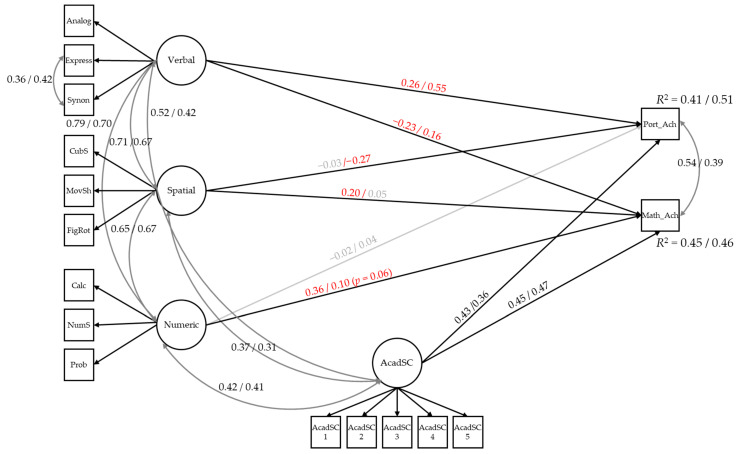
Multigroup structural model linking cognitive abilities and academic self-concept to Portuguese and mathematics achievement in third cycle and secondary education students. *Note.* AcadSC = academic self-concept; Port_Ach = Portuguese language achievement; Math_Ach = mathematics achievement. Mother’s educational level and student gender were included as covariates in the model, but their paths were omitted from the figure for visual clarity. Path coefficients are presented as 3rd cycle/secondary education. Red coefficients indicate statistically significant differences between groups based on multigroup comparisons, whereas grey coefficients indicate non-significant effects. All coefficients represent standardised estimates within each educational level.

Figure 2 presents the multigroup structural equation model in which verbal, spatial, and numeric abilities, along with academic self-concept, predict Portuguese and mathematics achievement for third cycle and secondary education students. As expected, verbal ability is positively associated with Portuguese language achievement (β = 0.26 for third cycle students, β = 0.55 for secondary students), spatial and numeric abilities are positively associated with mathematics achievement (spatial ability: β = 0.20 for third cycle students, β = 0.05, *p* = .294 for secondary students; numeric ability: β = 0.36 for third cycle students, β = 0.10, *p* = .051 for secondary students), and academic self-concept is positively related to both Portuguese language achievement (β = 0.43 for third cycle students, β = 0.36 for secondary students) and mathematics achievement (β = 0.45 for third cycle students, β = 0.47 for secondary students). In addition to these expected relations, verbal ability shows associations with mathematics achievement that differ in direction between third cycle (β = −0.23) and secondary education students (β = 0.16), whereas spatial ability is negatively related to Portuguese language achievement in both groups, but particularly in secondary education students (β = −0.27).

The proportion of explained variance in mathematics achievement was very similar across groups (third cycle: *R*^2^ = 0.45; secondary education: *R*^2^ = 0.46). In contrast, for Portuguese achievement, the explained variance was higher in secondary education (*R*^2^ = 0.51) than in the third cycle (*R*^2^ = 0.41).

A multigroup Wald test was conducted to examine whether the regression paths from cognitive abilities (verbal, numeric, and spatial), and academic self-concept to Portuguese and mathematics differed between students in the third cycle and secondary education. The overall test was statistically significant (χ^2^(8) = 45.45, *p* < .001), indicating that the set of predictive effects was not equivalent across groups. For Portuguese performance, significant group differences were observed in the effects of verbal and spatial abilities. Verbal ability showed a stronger positive effect in the secondary group compared to third cycle students (*d_p1q1* = −0.139, *p* = .003). Spatial ability also differed significantly across groups (*d_p3q3* = 0.103, *p* = .001), with a substantially stronger negative effect in the secondary group, while the effect in third cycle students was small and non-significant. No significant differences emerged for numeric ability or academic self-concept. For mathematics performance, significant between-group differences were found for verbal, numeric, and spatial abilities. Verbal ability again showed a stronger effect in the secondary group (*d_p7q7* = −0.168, *p* < .001). In contrast, numeric ability (*d_p8q8* = 0.086, *p* = .005) and spatial ability (*d_p9q9* = 0.066, *p* = .023) displayed stronger effects in the C3 group than in the secondary group. No significant group differences were detected for the effect of academic self-concept on mathematics achievement.

Table 3 summarises the effects of gender and mother’s education on achievement, cognitive abilities, and academic self-concept separately for the third cycle and secondary education groups (using this study’s gender coding, higher values correspond to girls). Gender showed significant associations with both achievement outcomes, with higher Portuguese and mathematics achievement for female students in both cycles. For cognitive abilities, there were no significant gender differences in verbal ability in either cycle, whereas gender effects were evident for spatial and numeric abilities in both cycles, in the same direction. Specifically, boys scored higher on spatial ability in both cycles and higher on numeric ability in both cycles, with the numeric effect being stronger in secondary education. Academic self-concept also differed by gender, with boys reporting higher academic self-concept in both cycles. Mother’s educational level showed consistently positive effects across all outcomes in both cycles. Higher maternal education was associated with higher Portuguese and mathematics achievement, higher cognitive abilities, and higher academic self-concept, with effects generally stronger in the third cycle than in secondary education for verbal, numeric, spatial, and self-concept aspects.

## 4. Discussion

This study examined the extent to which broad cognitive abilities (verbal, numerical, and spatial) and academic self-concept jointly predict achievement in Portuguese language and mathematics, and whether these relations differ between the third cycle of basic education and secondary education. The multigroup structural model was well fitted and showed that these psychological factors explain a substantial proportion of achievement variance. In mathematics, the set of predictors accounted for roughly 45% of the variance in grades in both school cycles, whereas in Portuguese, the explained variance was lower in the third cycle (about 40%) than in secondary education (about 50%). Overall, these results support the view that academic success reflects a combined contribution of cognitive resources and motivational beliefs (Deary et al., 2007; Steinmayr et al., 2018). Across educational levels and domains, academic self-concept emerged as the strongest and most consistent predictor of achievement. Self-concept becomes more stable and more closely linked to the evaluative feedback that students receive throughout their schooling, which can strengthen its predictive role throughout adolescence. This result converges with evidence showing that self-concept explains meaningful variance in school outcomes beyond cognitive ability and other individual differences (Lavrijsen et al., 2022) and remains a relevant correlate of achievement across adolescence (Perinelli et al., 2022; Sticca et al., 2023). The use of teacher-assigned grades may also have contributed to the robustness of this association, because grades capture ongoing classroom feedback and evaluative processes that are closely tied to students’ competence perceptions (Marsh et al., 2016).

The cognitive predictors of achievement showed clear differentiation across school cycles, consistent with the Cattell–Horn–Carroll (CHC) framework (J. B. Carroll, 1993; Schneider & McGrew, 2018). Verbal ability, aligned with CHC comprehension knowledge (Gc), had a stronger positive effect in secondary education on Portuguese achievement and, to a lesser extent, mathematics. This pattern is in line with CHC syntheses showing that Gc becomes increasingly influential as students’ progress and curricula place greater demands on vocabulary, background knowledge, and text-level comprehension (Hajovsky et al., 2025; McGrew & Wendling, 2010). It is also reflected in Portuguese language studies linking verbal cognitive abilities to reading comprehension (Joly & Dias, 2012; Lima & Santos, 2017). The heightened role of verbal ability for Portuguese in secondary education relative to the third cycle likely reflects the greater complexity of tasks at this level, where verbal comprehension is central to interpreting literary texts, analysing argumentative texts, and producing critical commentary, as specified in the curriculum (Direção-Geral da Educação, 2018a, 2018b). The stronger verbal association with mathematics at later stages may likewise reflect greater linguistic demands in problem solving and conceptual reasoning, consistent with evidence that Gc contributes to mathematics when tasks are language-loaded (Caemmerer et al., 2018; Zaboski et al., 2018).

In contrast, numerical (Gq) and spatial (Gv) abilities were more predictive of mathematics achievement in the third cycle. This result aligns with meta-analytic evidence that quantitative reasoning and visuospatial processing are meaningfully associated with mathematical performance, particularly for calculation and geometry-related content (Caemmerer et al., 2018; Zaboski et al., 2018). Developmentally, this may reflect a relatively greater emphasis in earlier curricula on procedural fluency and representational or spatial supports (e.g., geometric tasks that rely on visualisation). It is also consistent with work suggesting that specific cognitive skills may map differently onto mathematics outcomes depending on the content and cognitive mediation required by the tasks (Delgado & Prieto, 2004).

Two findings warrant additional comment. First, the negative association between verbal ability and mathematics achievement in the third cycle was unexpected. One possibility is a suppression effect arising from modelling multiple correlated broad abilities simultaneously, such that variance in verbal performance unrelated to mathematics-relevant reasoning becomes negatively weighted once quantitative ability and self-beliefs are controlled. This interpretation aligns with methodological work showing that the estimated effects of broad abilities can change substantially depending on how general and specific variance is modelled (Benson et al., 2016; Kan et al., 2024; Zaboski et al., 2018). In light of this, future studies could explore alternative CHC-consistent specifications (e.g., models separating general ability variance more explicitly) consistent with recent discussions in this literature (Lemos et al., 2020, 2025).

Second, spatial ability showed a negative relation with Portuguese achievement that was stronger in secondary education. From a CHC perspective, this may reflect the decreasing relevance of visuospatial processing for advanced literacy tasks that increasingly depend on verbal reasoning and discourse-level comprehension (Hajovsky et al., 2025; McGrew & Wendling, 2010). A complementary contextual explanation is that in secondary education, students’ curricular pathways and interests become more differentiated; greater investment in humanities and social science tracks may co-occur with lower engagement in spatial tasks, while STEM-oriented students may focus their effort on mathematics rather than language outcomes. This interpretation is consistent with evidence linking self-concept, interests, and educational choices during adolescence (Sinclair et al., 2019) and research on differentiated pathways in STEM trajectories (A. R. Costa et al., 2025). Research suggests that cognitive differentiation and crystallisation during adolescence develop in response to learning experiences, as well as personal interests and investment traits (Ackerman, 1996; Cattell, 1987). Contemporary research reinforces the dynamic interaction between investment traits, such as the need for cognition, and cognitive abilities (Scherrer et al., 2024), along with the role of broader cognitive–emotional maturation in shaping how adolescents learn and engage (Tarek, 2025). These developmental processes tend to become more differentiated with age and schooling, especially when students are required to make vocational decisions, as occurs in Portugal at the end of basic education (9th grade).

From an educational standpoint, the findings suggest that teaching and support practices may be more effective when aligned with the cognitive demands characteristic of each stage of schooling. In the third cycle, the prominence of numeric and visuospatial abilities points to the value of reinforcing foundational quantitative reasoning while making mathematical representations explicit (e.g., supporting students in translating between symbols, diagrams, and verbal statements). At this stage, structured opportunities to articulate solution steps and justify procedures may help connect verbal explanations to quantitative meaning, supporting comprehension without overburdening students with the more formal academic language demands of later schooling. In secondary education, by contrast, the stronger contribution of verbal and numeric abilities is consistent with the increased curricular emphasis on abstraction, multi-step reasoning, and the interpretation of complex problem statements, where academic language and conceptual precision become more central to mathematical performance. This developmental shift aligns with international frameworks (OECD, 2024, 2025), emphasising the integration of language-mediated reasoning and higher-order problem solving as schooling progresses. Across both cycles and achievement domains, the robust role of academic self-concept further suggests that instructional support should be paired with classroom practices that strengthen students’ perceived competence, such as formative feedback focused on progress and strategy use, opportunities for successful engagement with appropriately challenging work, and teacher messages that emphasise that ability can improve with practice and support.

Regarding sociodemographic covariates, male students tended to obtain higher scores on spatial tasks in both school cycles and higher numerical scores in secondary education. These patterns align with reviews documenting relatively reliable gender differences in spatial ability, particularly for tasks involving mental rotation and spatial visualisation (Bartlett & Camba, 2023; Hegarty, 2018), and evidence that gender differences in numerical outcomes can be small and context-dependent (Halpern, 2013). In terms of school achievement, female students showed higher performance in Portuguese, a pattern consistent with meta-analytic evidence of girls’ advantage in language-related achievement and work highlighting higher engagement and task persistence among female students (M. Carroll, 2023; Johannsen et al., 2024; Voyer & Voyer, 2014).

Mother’s educational level was positively related to cognitive performance, academic self-concept, and achievement in both Portuguese and mathematics. This replicates the robust literature linking parental education and socioeconomic resources to more cognitively stimulating learning environments and better academic outcomes (Harding, 2015; Magnuson, 2007; Tan et al., 2019). The present results are also consistent with evidence from Portuguese samples showing the salience of maternal education for children’s cognitive performance (Alves et al., 2016).

Several limitations should be considered. First, educational level was analysed cross-sectionally, meaning that developmental interpretations must be made with caution. The differences observed between educational levels reflect cross-sectional contrasts rather than within-student developmental change. Longitudinal designs will therefore be necessary to track changes within the same students as they transition into secondary education.

Second, academic achievement was operationalised through teacher-assigned grades, which were subsequently standardised within level. Although this approach increases comparability across different grading systems, it does not remove potential variability in grading practices between educational cycles and schools. Although standardised external assessments are part of the national evaluation system (final examinations at the end of grade 9 and national examinations at the end of grades 11–12), it was not possible to obtain these scores for the present sample due to the time lag between the administration of our assessment protocol and the later release of external results, combined with limited access to these data in participating schools. As a result, academic achievement relied exclusively on teacher-assigned grades. These grades are ecologically valid indicators of school performance but may also reflect differences in assessment practices across schools.

Third, the magnitude of the associations examined may depend on how achievement is measured. Intelligence tends to show stronger associations with standardised achievement tests, whereas teacher-assigned grades often capture a broader constellation of influences. Such influences include noncognitive and motivational factors, such as self-regulation and academic self-beliefs, which typically display stronger links with grades (Borghans et al., 2016; Duckworth et al., 2012; Lavrijsen et al., 2022). Consequently, relying on school grades in the present study may have amplified the observed associations between academic self-concept and achievement, and these results may not fully generalise to standardised test outcomes.

Fourth, academic self-concept was assessed as a general perception of school competence. This choice ensured parsimony in the structural model and aligned with our focus on broader cognitive–motivational patterns across educational levels rather than domain-specific associations. Because we used a global measure, it may have overlooked distinct verbal versus mathematical self-concept and may therefore have underestimated domain-specific associations with Portuguese and mathematics achievement. Future studies should incorporate domain-specific self-concept measures to examine whether verbal and mathematical self-beliefs show distinct relations with corresponding academic outcomes.

Finally, because the BAC-AB test versions differ across educational cycles (even with anchor items), and because relations between cognitive test performance and achievement can vary by grade level (Lemos & Almeida, 2019), replication using multiple cognitive indicators and additional achievement measures would strengthen the developmental conclusions.

## 5. Conclusions

The findings indicate that adolescent achievement in Portuguese and mathematics is jointly associated with broad cognitive abilities and academic self-concept across the third cycle of basic education and secondary education. Academic self-concept emerged as the most robust and consistent predictor across curricular domains and educational levels. Its stronger role in later schooling may reflect the increasing stability of self-beliefs throughout adolescence and the cumulative impact of evaluative feedback in contexts where learning demands intensify and grading becomes more differentiated.

With regard to cognitive predictors, the results point to a developmental shift consistent with the CHC framework. Verbal ability (Gc) became more influential in secondary education, showing the strongest contribution to Portuguese achievement and with a growing role in mathematics. In contrast, numeric (Gq) and spatial (Gv) abilities were more predictive earlier in schooling, particularly for mathematics in the third cycle. These patterns align with the idea that instruction should be developmentally sensitive. In earlier years, it may be beneficial to reinforce quantitative practice and use visuospatial supports; later, as curricular tasks become more complex, greater emphasis can be placed on comprehension, abstraction, and language-based reasoning, including in mathematical problem solving. In this sense, strengthening foundational competencies in basic education may help students better navigate the transition from numerical skills and concrete representations toward more abstract, language-mediated, mathematical reasoning.

Ultimately, the results suggest that cognitive abilities matter, but they are not sufficient on their own. Students also need a strong sense of competence in order to fully translate their skills into academic success. In this regard, academic self-concept appears to function as a motivational resource that supports engagement with learning demands and persistence through challenges. This underscores the importance of assessment and feedback practices that communicate progress and effective strategies, while avoiding messages that may inadvertently reinforce negative self-perceptions in students. Feedback that is constructive, specific, and growth-oriented may help sustain more adaptive self-beliefs and enable students to make effective use of their cognitive abilities.

## Figures and Tables

**Table 1:** Reliabilities, descriptive statistics, and Pearson correlations among study variables.

	α	*M*	*SD*	Port	Math	Verbal	Spatial	Numeric	Acad SC	Gender	Moth_Ed
Port	-	3.05/13.09	0.81/2.73	1	0.61 **	0.41 **	0.11 **	0.23 **	0.43 **	0.22 **	0.16 **
Math	-	2.99/12.17	1.03/3.82	0.61 **	1	0.34 **	0.24 **	0.31 **	0.47 **	0.12 **	0.15 **
Verbal	0.75	34.87/40.65	10.18/9.14	0.38 **	0.32 **	1	0.43 **	0.47 **	0.34 **	−0.02	0.17 **
Spatial	0.73	30.95/34.29	9.29/9.06	0.23 **	0.35 **	0.47 **	1	0.55 **	0.28 **	−0.19 **	0.12 **
Numeric	0.77	33.88/38.56	13.92/12.09	0.33 **	0.45 **	0.58 **	0.59 **	1	0.35 **	−0.20 **	0.15 **
Acad_SC	0.78	2.70/2.70	0.52/0.48	0.45 **	0.49 **	0.40 **	0.33 **	0.40 **	1	−0.10 **	0.19 **
Gender	-	-	-	0.20 **	0.03	0.06 *	−0.14 **	−0.09 **	−0.11 **	1	-
Moth_Ed	-	-	-	0.18 **	0.16 **	0.24 **	0.20 **	0.24 **	0.26 **	-	1

*Note.* α—Cronbach’s alpha, Port = Portuguese language grades, Math = mathematics grades, Acad SC = academic self-concept, Moth_Ed = mother’s education. For means and standard deviations, the first value refers to 3rd cycle students and the second to secondary school students. Values below the diagonal are correlations among constructs for 3rd cycle students and values above the diagonal are correlations among constructs for secondary school students. * *p* < .05. ** *p* < .01.

**Table 2:** Measurement invariance results for cognitive abilities and academic self-concept.

	Χ^2^	*df*	*p*	CFI	TLI	RMSEA with 95% CI	SRMR	ΔCFI	ΔRMSEA
BAC—Unidimensional									
Configural	153.1	36	<.001	0.982	0.965	0.046, [0.039, 0.054]		-	-
Metric	215.3	44	<.001	0.974	0.958	0.051, [044, 0.058]		0.008	−0.005
Scalar	328.3	52	<.001	0.958	0.942	0.059, [0.053, 0.065]		0.016	−0.008
BAC—Multidimensional									
Configural	368.9	46	<.001	0.951	0.924	0.068, [0.062, 0.075]	0.039	-	-
Metric	422.8	52	<.001	0.944	0.923	0.063, [0.063, 0.075]	0.055	0.007	0.005
Scalar	436.4	58	<.001	0.943	0.929	0.066, [0.06, 0.071]	0.061	0.001	−0.003
AcadSC									
Configural	52.7	10	<.001	0.982	0.963	0.056, [0.042, 0.072]	0.022	-	-
Metric	59.0	14	<.001	0.981	0.972	0.049, [0.036, 0.062]	0.03	0.001	0.007
Scalar	78.1	18	<.001	0.974	0.971	0.050, [0.039, 0.061]	0.029	0.007	−0.001

**Table 3:** Effects of gender and mother’s education on academic achievement, cognitive abilities, and academic self-concept by school level.

	Port	Math	Verbal	Spatial	Numeric	Academic Self-Concept
Gender						
3rd Cycle	0.20 ***	0.16 ***	0.06	−0.16 ***	−0.09 **	−0.12 ***
Secondary	0.22 ***	0.18 ***	−0.04	−0.19 ***	−0.22 ***	−0.09 **
Mother’s Education						
3rd Cycle	0.08 **	0.06 *	0.30 ***	0.25 ***	0.25 ***	0.29 ***
Secondary	0.07 **	0.10 ***	0.20 ***	0.12 ***	0.15 ***	0.21 ***

*Note.* Port = Portuguese language achievement, Math = mathematics achievement. * *p* < .05. ** *p* < .01. *** *p* < .001.

## Data Availability

The data presented in this study are available on request from the corresponding author due to privacy and confidentiality restrictions. Data sharing is conducted in accordance with the consent provided by participants, and the publication of the data does not compromise participant anonymity or violate local data protection regulations.
